# Extraction of Cannabinoids and Terpenes from Hemp Flowers and Leaves (*Cannabis sativa* L., Futura 75): Chemical Profiling and Evaluation of Anticancer Properties

**DOI:** 10.3390/molecules30061325

**Published:** 2025-03-15

**Authors:** Monika Haczkiewicz, Marta Świtalska, Jacek Łyczko, Magdalena Pluta, Joanna Wietrzyk, Anna Gliszczyńska

**Affiliations:** 1 Department of Food Chemistry and Biocatalysis, Wrocław University of Environmental and Life Sciences, Norwida 25, 50-375 Wrocław, Poland; monika.haczkiewicz@upwr.edu.pl (M.H.); jacek.lyczko@upwr.edu.pl (J.Ł.); magdalena.pluta@upwr.edu.pl (M.P.); 2 Department of Experimental Oncology, Ludwik Hirszfeld Institute of Immunology and Experimental Therapy, Polish Academy of Sciences, Weigla 12, 53-114 Wrocław, Poland; marta.switalska@hirszfeld.pl (M.Ś.); joanna.wietrzyk@hirszfeld.pl (J.W.)

**Keywords:** cannabinoids, terpenes, solvent extraction, food safety, anticancer activity

## Abstract

This study investigated efficient extraction methods for cannabinoids and terpenes from the above-ground parts of Futura 75, focusing on two techniques: pressurized extraction and magnetic stirrer-assisted extraction. The effects of solvent type, temperature, time, and pressure were evaluated using five organic solvents and two binary solvent systems. Cannabinoid profiles of obtained extracts were analyzed using gas chromatography coupled with mass spectrometry (GC-MS), while terpene profiles were characterized through solid-phase microextraction (SPME) combined with GC-MS. Next, two selected extracts with the highest content of cannabinoid and terpene fractions (Futu1 and Futu2) were tested for antiproliferative activity toward cancer cell lines (MV4-11, AGS, HT-29, MDA-MB-468, MCF-7) and their cytotoxicity was evaluated on non-tumorigenic MCF-10A cells. Extract Futu1 contained 51.57% cannabinoids, 9.8% monoterpenes, and 90.2% sesquiterpenes in the terpene fraction. Futu2 exhibited a higher proportion of monoterpenes in the terpene fraction (19.6% monoterpenes and 80.4% sesquiterpenes) and consisted of 49.49% cannabinoids. Both extracts exhibited higher selectivity for cancer cells over non-tumorigenic cells, with Futu2 demonstrating stronger antiproliferative properties. Interestingly, lower concentrations of extracts and tested free, single cannabinoids stimulated the growth of leukemia (MV4-11) and breast cancer (MDA-MB-468) cell lines while their higher concentrations suppressed proliferation.

## 1. Introduction

*Cannabis sativa* L., commonly known as hemp, belongs to the *Cannabaceae* family, which includes two primary cannabis varieties: hemp (*Cannabis sativa* L. var. *sativa*) and marijuana (*Cannabis sativa* L. var. *indica*). According to Polish regulations [1], in first-grade hemp, the combined content of ∆^9^-tetrahydrocannabinol (THC) and tetrahydrocannabinolic acid (THCA) must not exceed 0.3% on a dry-weight basis. In contrast, in narcotic hemp, the concentration of this compound can reach up to 20% (*w*/*w*) [2]. Cannabis strains can be classified into five chemotypes: type I—narcotic plants with a high ∆^9^-THC content, type II—medicinal hemp containing Δ^9^-THC and cannabidiol (CBD) in various ratios, type III—fiber hemp with a high CBD content and low Δ^9^-THC (<0.3% *w*/*w*), type IV—fiber hemp containing cannabigerol (CBG), and type V—with plants mostly devoid of cannabinoids, primarily used in the textile industry [3].

Futura 75 is one of approximately 70 hemp varieties approved for commercial production and distribution in the European Union [4]. Over the past few decades, this monoecious cultivar of French origin has been the most widely cultivated hemp variety in Italy [5]. It is characterized by a low content of ∆^9^-THC and is commercially used in food preparation, particularly regarding seeds and fiber.

Interest in hemp-based products has been steadily increasing each year. The most popular food products are produced from seeds, hemp flour, cannabinoid oils, and extracts [6]. According to the Regulation of the European Parliament and Council (EU), hemp seeds can be incorporated into food, whereas other hemp parts and extracts are classified as “novel foods”. Hemp and its extracts are also gaining growing consumer attention for their therapeutic properties and health-promoting benefits. This trend increased after 2018 when the U.S. Food and Drug Administration (FDA) approved Epidiolex (a CBD oral solution) as the medication for epilepsy.

In hemp, 750 natural compounds have been identified. The primary ones include amino acids, fatty acids, and steroids, while the secondary metabolites consist of phytocannabinoids, flavonoids, terpenoids, lignans, and alkaloids [7]. Phytocannabinoids occur in glandular hairs and, from a chemical point of view, they are terpenophenolic compounds (isoprenylated polyketides) containing 19 to 23 carbon atoms, depending on the length of the alkyl side chain. These include the following acids: cannabigerolic acid (CBGA), which serves as a precursor of ∆^9^-tetrahydrocannabinolic acid (∆^9^-THCA); cannabidiolic acid (CBDA); and cannabichromenic acid (CBCA). In most hemp varieties, these compounds exist in their acidic form and are sensitive to light, oxygen, and temperature. Under the influence of these factors, a decarboxylation reaction takes place, giving the respective products: cannabigerol (CBG), ∆^9^-tetrahydrocannabinol (∆^9^-THC), cannabidiol (CBD), and cannabichromene (CBC), which often have higher biological activity than the initial acidic forms [8].

Over 140 cannabinoids have been identified in cannabis so far. Cannabigerol (CBG) has a wide range of biological activities, including anti-inflammatory [9], antibacterial [10], and antifungal activity [11]. It also plays a role in the regulation of redox balance and gives a neuromodulatory effect. Therefore, this compound can be used in the treatment of patients requiring multidirectional pharmacotherapy [12]. Psychoactive THC has antiemetic [13], anti-inflammatory [14], and analgesic effects and is used in the treatment of neuropathy and chronic pain [15]. Cannabidiol (CBD) is present in a much higher concentration in fibrous hemp and, as a non-psychoactive substance, has several therapeutic properties used in pharmacology. The results of numerous studies indicate that CBD exhibits the following effects: anticancer, analgesic, neuroprotective, antiemetic, anticonvulsant, anti-inflammatory, and antispasmodic. Additionally, it is used in the treatment of epilepsy and nausea [16]. Cannabichromene (CBC) reduces inflammation [17], provides analgesic effects, inhibits prostaglandin synthesis in vitro, and demonstrates strong antibacterial as well as mild antifungal activity [18,19].

The terpenes present in cannabis are of equally important therapeutic importance. They have been granted Generally Recognized as Safe (GRAS) status by the FDA and can be used as flavoring or nutritional additives. The content of terpenes and cannabinoids in cannabis is typically positively correlated [20]. The most common monoterpenes in cannabis include myrcene, α-pinene, limonene, and linalool. Among sesquiterpenes, the highest concentrations have been recorded for β-caryophyllene, bisabolol, and (*E*)-β-farnesene as well as α-humulene, which appears to be present in most cannabis varieties [21]. To date, approximately 200 terpenes have been identified in cannabis [22].

Studies show that the compounds that make up the constituents in cannabis in the mixture exhibit more potency [23]. It has been confirmed that terpenes enhance the therapeutic effect of cannabinoids [24]. The final content and profile of biologically active compounds in cannabis-based products are influenced by many factors, such as hemp variety, harvest time, and external conditions affecting plant growth. From a technological perspective, the method of processing the raw material and the extraction technique used to isolate active compounds from the plant material are also of key importance. The biological effectiveness of terpenes has been observed only when their concentration in the hemp extract exceeds 0.05% *v*/*w*.

The extraction of oil containing not only cannabinoids but also naturally occurring terpenes in hemp is highly challenging. Therefore, most often, terpenes are added to oils and other hemp products, which, of course, is not a reflection of the natural composition [25]. There is a need to develop sustainable hemp extraction processes that combine high efficiency and the safe recovery of biologically active compounds without causing their degradation or loss [26]. Differences in extraction methods determine the profile of active compounds in extracts and, thus, differences in their biological effects and effectiveness [27]. Moreover, the parameters of the extraction significantly impact the stability and quality of the final preparation, directly affecting its safety and usability [28].

Therefore, the purpose of this research work was to optimize the extraction parameters for cannabinoid and terpene fractions from the Futura 75 hemp variety, cultivated under certified conditions in Poland. The extraction process utilized solvents approved for food production, followed by a comprehensive analysis of the extracted product profile. For the extract with the highest content of both fractions, biological tests were carried out in an in vitro model to evaluate its safety toward non-tumorigenic cells and its cytotoxic effect on selected cancer cell lines representing some of the most common cancer types worldwide.

## 2. Results and Discussion

### 2.1. Plant Material and Extraction

To obtain an extract rich in cannabinoids and terpenes, it was crucial to analyze the effects of extraction parameters such as the type of organic solvent, temperature, time, and pressure on the composition of the products isolated in the process. For the extraction of cannabinoids and terpenes from the Futura 75 hemp variety, five types of organic solvents (acetone, methanol, ethanol, isopropanol, hexane) were used, along with two binary solvent mixtures hexane/isopropanol (7:3, *v*/*v*) and hexane/ethanol (7:3, *v*/*v*). These binary systems were selected based on literature data, which indicated that a combination of nonpolar and polar solvents could improve the efficiency of cannabinoid and terpene extraction [29]. Processes of extractions were performed using two methods: pressurized extraction and magnetic stirring at temperatures of 25 °C and −55 °C. Farokhi and colleagues demonstrated that ethanol extraction at low temperatures ranging from 0 °C to −80 °C enables the isolation of cannabinoids and terpenoids while reducing the presence of waxes and chlorophyll [30].

The results showed that the highest yield for pressurized extraction (0.46 g) was achieved using methanol as the solvent at 25 °C and a pressure of 2 bar. These findings align with literature data indicating that the methanol-based extraction of bioactive compounds from hemp yields higher efficiencies compared to ethanol and other organic solvents [27] (Table S1, Supplementary Materials). Contrary to initial expectations, the use of binary solvent systems did not significantly enhance the extract yield, as shown in Table S1 (Supplementary Materials). Furthermore, applying 2 bar of pressure during extractions with acetone, hexane, and binary solvent mixtures resulted in lower yields compared to processes conducted at 1 bar.

In the second experimental approach, the extraction process was conducted using a magnetic stirrer (180 rpm) for 10, 20, and 40 min at temperatures of 25 °C and −55 °C, using the same organic solvents as in the pressurized method. The results are summarized in Table S2 (Supplementary Materials). Similar to the pressurized method, the highest extraction yield was achieved with methanol at 25 °C, and the yield increased with longer extraction times.

The extracts obtained from both methods were subjected to a winterization process to remove waxes (Section 3.2.2). Based on the results (Tables S1 and S2, Supplementary Materials), it was observed that, regardless of the extraction method, the largest mass reduction of 48% after winterization was recorded for extracts obtained using hexane at 25 °C. These findings confirm that decreasing the polarity of the organic solvents used in hemp extraction increases the wax content in the extracts, which is subsequently removed during the winterization process [31].

### 2.2. Analysis of Cannabinoid Profiles of Extracts

In the next stage of this study, the cannabinoid profiles of extracts obtained through magnetic stirrer-assisted extraction and pressurized extraction at 1 and 2 bar were analyzed using gas chromatography coupled with mass spectrometry (GC-MS). For pressurized extraction using less polar solvents such as acetone, hexane, and their mixtures, the highest cannabinoid fractions were achieved at the lower pressure of 1 bar, which allowed for a longer contact time between the solvents and the raw material bed. Conversely, for ethanol, methanol, and isopropanol, higher cannabinoid extraction efficiencies were observed at the higher pressure of 2 bar. The optimal pressure values for each solvent were determined experimentally by analyzing the residual cannabinoid content in the post-extraction material, ensuring that it was below 5.7% relative to the starting material.

Table 1 presents the cannabinoid fractions in extracts obtained via pressurized extraction (at 1 or 2 bar) at temperatures of 25 °C and −55 °C. Due to the significant fragmentation of the plant material and concerns about the undesirable extraction of chlorophyll, a temperature of −55 °C was used for extraction. Moreover, according to the literature, using the mentioned solvents at extraction temperatures between −60 °C and −40 °C on a large scale allows for extracts of higher purity to be obtained [32].

Across all variants using different solvents, the cannabinoid profiles varied and ranged from 39.85% to 51.57%. The highest concentration of the cannabinoid fraction from the Futura 75 variety was achieved through pressurized extraction (1 bar) using a hexane/ethanol solvent mixture at a 7:3 volumetric ratio and a temperature of 25 °C. Although the extraction yield under these parameters was not the highest (yielding only 0.29 g of extract, as shown in Table S1 in the Supplementary Materials), the extract contained the highest cannabinoid concentration of 51.57% (Table 1).

For comparison, in the variant with the highest extraction yield (0.46 g of extract obtained using methanol at 2 bar and 25 °C), the cannabinoid fraction was the lowest, at 42.01% (Table 1).

**Figure 1:**
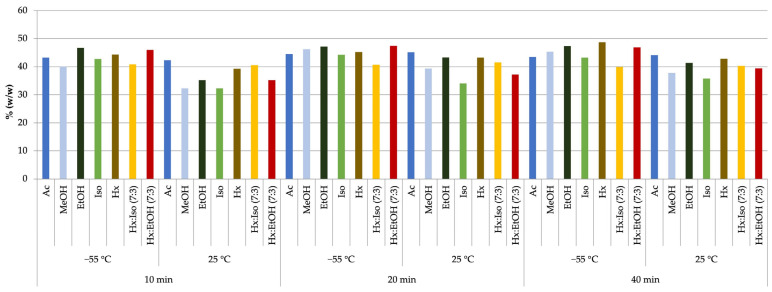
Cannabinoid content of extracts from the Futura 75 cultivar obtained by magnetic stirrer extraction with organic solvents at 25 °C and −55 °C.

In extracts obtained through magnetic stirrer-assisted extraction, the highest cannabinoid fraction (48.76%) was achieved using hexane for 40 min at −55 °C (Figure 1). However, the yield of the extract in this case was significantly lower (0.20 g) compared to the extraction variant using methanol at 25 °C for 40 min, where the highest extract mass of 0.45 g was obtained, though the cannabinoid content was below 40%.

Additionally, it was observed that prolonging the extraction time with methanol, ethanol, or acetone did not significantly increase the cannabinoid content. In contrast, increasing the extraction temperature led to a reduction in cannabinoid levels and an increase in undesirable components such as waxes and triacylglycerols [32].

### 2.3. Terpene Organic Compound Profiles of Extracts

**Figure 2:**
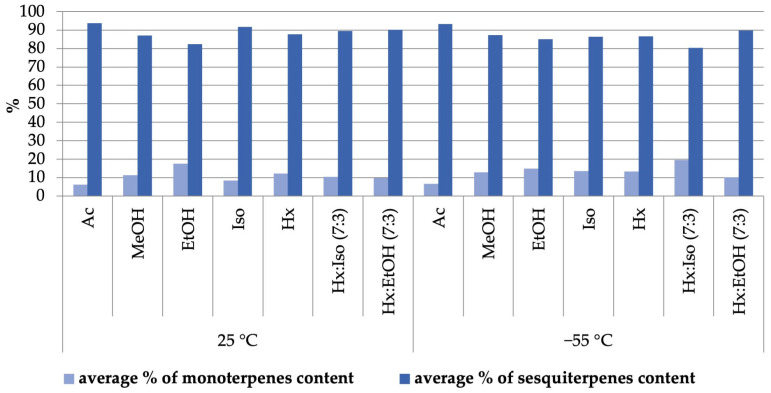
The influence of organic solvent on the efficiency of extraction of terpene compounds during pressure extraction from the Futura 75 hemp variety.

The terpene profiles of extracts obtained through pressurized extraction were analyzed using solid-phase microextraction (SPME) combined with GC/MS. The lowest monoterpene-to-sesquiterpene ratio was observed in extracts obtained using acetone at 25 °C. Conversely, the highest monoterpene content relative to sesquiterpenes was achieved using a hexane/isopropanol mixture (7:3 *v*/*v*) at −55 °C. Namdar et al. highlighted in their study that a high extraction efficiency for both cannabinoids and terpenes can be achieved using a combination of polar and nonpolar solvents [29]. This observation aligns with the results obtained in our study (Figure 2).

**Figure 3:**
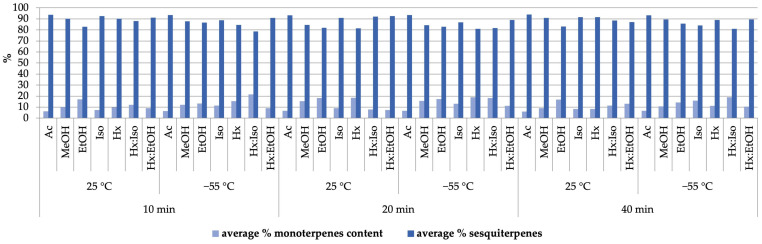
Influence of selected parameters: type of organic solvent, temperature, and time (10, 20, and 40 min) on the terpenes profile in the extracts obtained from Futura 75 by extraction performed by a magnetic stirrer.

The analysis of the terpene fraction profile in extracts obtained using magnetic stirrer-assisted extraction yielded results analogous to those observed for the previous extraction method. The lowest monoterpene-to-sesquiterpene ratio was identified in extracts obtained with acetone at 25 °C. Conversely, the highest monoterpene content relative to sesquiterpenes was observed in extracts obtained using the same binary solvent mixture (hexane/isopropanol, 7:3 *v*/*v*) as in the pressurized extraction at a temperature of −55 °C (Figure 3).

In the next stage of this study, the identification of terpene compounds present in the extracts was based on two independent methods: comparing the obtained mass spectra with the NIST 20 (National Institute of Standards and Technology) and FFNSC 3.0 (Flavours and Fragrances of Natural and Synthetic Compounds 3.0) databases and comparing the calculated retention indices’ LRIs using a retention index calculator with the values provided in the NIST 20 and FFNSC 3.0 databases. A total of 59 compounds were identified in the extracts obtained via both pressurized extraction and magnetic stirrer-assisted extraction, as presented in Table 2 below.

Terpene profiles are unique to each cannabis variety [33] and change throughout a plant’s growth cycle. Sesquiterpene concentrations are higher during the early growth stages, while monoterpene concentrations increase over time [26]. After harvesting and drying the plant material, the terpene and terpenoid content decreases due to the volatility of these compounds. The prolonged storage of raw material and temperatures above 60 °C used during solvent removal from the extract also negatively impact the concentration of these volatile substances. Hanus et al. reported that the monoterpene-to-sesquiterpene ratio in extracts obtained using solvents is lower than in essential oils [23].

Moreover, in the context of practical applications of cannabis extracts, it is crucial to understand and analyze the influence of the so-called “entourage effect”, referring to the impact of other natural compounds, such as terpenoids, present in the extract on its biological activity [34]. Literature data indicate that plant-based formulations are more effective than individual compounds [35], particularly in the context of anticancer and anti-inflammatory activity [36]. Furthermore, terpenes and cannabinoids exhibit synergistic interactions, enhancing their anticancer effects through the activation of CB2 receptors [37]. Therefore, it is essential to optimize cannabis extraction methods to achieve the highest concentrations of both groups of compounds.

### 2.4. Cytotoxicity of Extracts Toward Selected Cell Lines

Two extracts, Futu 1 and Futu 2, were selected for the study of their antiproliferative activity against six human cancer cell lines: biphenotypic B myelomonocytic leukemia (MV4-11), lung carcinoma (A549), colon adenocarcinoma (HT-29), gastric adenocarcinoma (AGS), and breast carcinomas (MCF-7 (ER+) and MDA-MB-468 (TNBC)). Additionally, their cytotoxicity and potential safety for use were assessed on normal human breast epithelial cells (MCF-10A). This study aimed to determine the effectiveness of these extracts in inhibiting cancer cell growth while ensuring minimal toxicity to normal cells. The first extract, Futu 1, contained the highest amount of cannabinoid fraction (51.57%) among all tested extracts, and the terpene fraction consisted of 9.8% monoterpenes and 90.2% of sesquiterpenes. Futu 1 was obtained using pressurized extraction with the following process parameters: a pressure of 1 bar, a solvent mixture of hexane/ethanol (7:3, *v*/*v*), and a temperature of 25 °C. The second extract, Futu 2, was selected for its higher monoterpene content (19.6%) in the terpene fraction in comparison to sesquiterpenes (80.4%) and its 49.49% content of cannabinoids. This extract was also obtained via pressurized extraction, using a pressure of 1 bar, a solvent mixture of hexane/isopropanol (7:3, *v*/*v*), and a temperature of −55 °C. After 72 h of incubation of cancer and normal cells with cannabinoids and the extracts Futu 1 or Futu 2, antiproliferative activity was assessed using the MTT assay (for MV4-11) or the SRB assay. The percentage of cell growth inhibition was calculated and IC_50_ values were determined (Table 3).

All cannabinoids had IC_50_ in a similar range: 2.87–10.04 μg/mL against lung (A549), colon (HT-29), gastric (AGS), and breast (MCF-7, ER+) cancer cell lines. The most active compound was CBD, with the IC_50_ ranging between 2.87 and 3.74 μg/mL. The cannabinoids had less cytotoxicity against normal MCF-10A cells (IC_50_ of about 10–11 μg/mL) with a selectivity index (SI) higher than 1 but, in most cases, in the range of 1–3 (Table 4). Futu1 and Futu2 had slightly lower antiproliferative activity than cannabinoids against cancer cells, with the IC_50_ in the range of 7.31–15.41 μg/mL (Table 3). Plant extracts also had lower cytotoxicity against MCF-10A (58.0 and 31.8 μg/mL) but a much higher selectivity index (above 3) compared to free cannabinoids.

**Figure 4:**
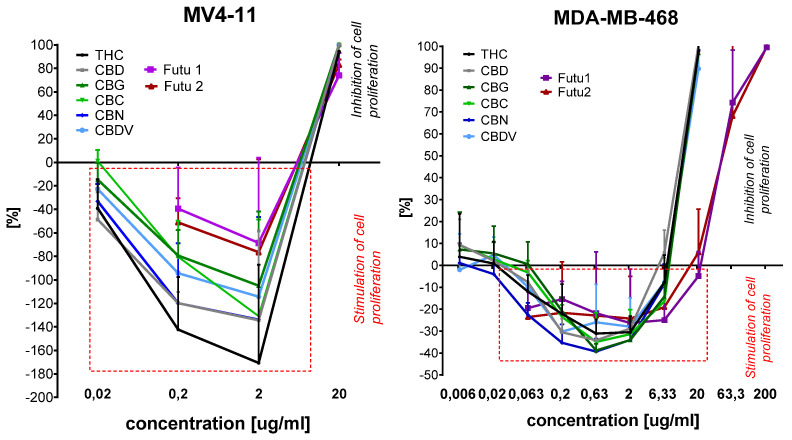
Activity of extract against MV4-11 leukemia and breast cancer MDA-MB-468 (TNBC) cell lines.

The analysis of the influence of cannabinoids and the Futu1 and Futu2 extracts on cancer cell growth showed that all of them in low concentrations stimulated the growth of leukemia MV4-11 and triple-negative breast MDA-MB-468 cells (Figure 4).

The cannabinoids at concentrations of 0.02–2 μg/mL and plant extracts at concentrations of 0.2–2.0 μg/mL strongly stimulated MV4-11 cell growth (the stimulation in Figure 4 is marked as (−) values; the inhibition of growth is marked as (+) values). At concentrations of 2 μg/mL, THC stimulated the growth of MV4-11 cells by 180% (compared to untreated control cells) and CBD, CBN, and CBC by 140%. At the same concentration, Futu1 and Futu2 stimulated growth by 70%. The inhibition of MV4-11 cell growth was observed only at the highest used concentrations. A similar observation was noticed in triple-negative breast cancer model MDA-MB-468 (Figure 4). All compounds at concentrations of 0.063–6.33 μg/mL stimulated cell growth. The highest stimulation of MDA-MB-468 growth was observed at a concentration of 0.63 μg/mL. Cannabinoids stimulated cell growth by 30–40% and Futu1 and Futu2 stimulated cell growth by 20–30% compared to untreated control cells (the stimulation in Figure 4 is marked as (−) values; the inhibition of growth is marked as (+) values). For concentrations higher than 6.33 μg/mL, the inhibition of cell proliferation was observed.

The effect of cell growth stimulation in some concentrations of the tested compounds was observed only for two of the used cell lines. These compounds stimulated growth only in the case of triple-negative breast cancer MDA-MB-468 cells and not ER+ breast cancer MCF-7 cells.

The complex effects of cannabinoids on cancer processes, as influenced by their concentration, molecular mechanisms, and cancer type, are well documented in the literature. Cannabinoids can both stimulate and inhibit the proliferation of cancer cells. For example, THC at low concentrations (nanomolar) may stimulate the growth of cancer cells, such as glioblastoma (U373-MG) and lung cancer (NCI-H292). This effect is attributed to the activation of CB1 receptor-dependent signaling pathways or receptor-independent mechanisms, such as the activation of TNF-alpha convertase (TACE/ADAM17) and the transactivation of the epidermal growth factor receptor (EGFR) [38]. Conversely, at higher concentrations (micromolar), THC exhibits antiproliferative and pro-apoptotic effects by inducing apoptosis in various cancer cell types. This mechanism often involves the activation of CB1/CB2 receptors and endoplasmic reticulum stress [39]. Cannabidiol (CBD), a non-psychoactive cannabinoid, predominantly exhibits antiproliferative activity through mechanisms independent of CB1 and CB2 receptors. CBD induces endoplasmic reticulum stress, activates TRPV2 receptors, modulates signaling pathways such as ERK and Akt, and promotes autophagy and apoptosis [40,41]. Additionally, CBD inhibits angiogenesis and the invasive capacity of cancer cells, making it a promising candidate for anticancer therapy.

Our findings, for the first time, indicate that natural extracts containing cannabinoid and terpenoid fractions may, at lower concentrations, also stimulate the proliferation of leukemia MV4-11 and breast cancer MDA-MB-468 cell lines. These results provide valuable insights into effective and safe therapeutic doses of the extracts for potential cancer treatment.

## 3. Materials and Methods

### 3.1. Chemicals and Solvents

All reagents and organic solvents were of analytical grade. Standards of cannabinoids: cannabidiol (CBD), cannabigerol (CBG), cannabinol (CBN), cannabichromene (CBC), and (–)-*trans*-Δ^9^-tetrahydrocannabinol (Δ^9^-THC) in the concentrations of 1 mg/mL in methanol were purchased from Merck (Darmstadt, Germany). Chloroform, methanol, ethanol (99% *v*/*v*), isopropanol, hexane, acetone, diphenylamine (>99%), and undecane-2-one (99.9%) were from Sigma-Aldrich (Steinheim, Germany).

### 3.2. Plant Material and Sample Processing

#### 3.2.1. Plant Material

The research material was the hemp variety *C. sativa* Futura 75 from a certified cultivation in Poland containing (–)-*trans* Δ^9^-tetrahydrocannabinol (Δ^9^-THC) at a level below 0.3% (*w*/*w*). The material consisted of above-ground parts of the plant in the form of 30 cm sections collected after the flowering phase, which were dried to a moisture content of 8% to 12% and stored at a temperature below 25 °C without access to light until the start of the research. Stems and seeds were removed from the plant material, which was next ground and sieved through a sieve with a mesh size of 1 mm. The material prepared in this way was then extracted.

#### 3.2.2. Low-Pressure Extraction (LPE)

The extraction of plant material was carried out in a specially made extractor equipped with a set of filters, where solvents were passed through the deposit under a pressure of 1 or 2 bar using nitrogen as the pressing gas. For comparative purposes, extraction was performed in parallel using a Heidolph Hei-Torque mechanical stirrer (Schwabach, Deutschland) set to 180 rpm for durations of 10, 20, and 40 min. Following each extraction, the plant material was separated by filtration in a vacuum funnel.

The extraction process was carried out with two temperature variants: 25 °C and −55 °C, with the use of solvents: acetone, methanol, ethanol, isopropanol, hexane, and mixtures: hexane with isopropanol and hexane with ethanol in a volume ratio of 7:3.

For low-pressure extraction, 200 mL of solvent, divided into two parts, was used for each 10 g sample of plant material. The first part of the solvent was recycled for re-extraction and the second part was passed through the bed. The solvents from the extracts were evaporated in a vacuum dryer (Goldbrunn 450). A single dose of 200 mL of solvent was used per 10 g of plant material for extraction using a mechanical stirrer. Each trial was performed in three repetitions.

To remove the waxes from the extracts, a winterization process was carried out consisting of dissolving the extracts in ethanol (99% *v*/*v*) at 50 °C for 20 min, cooling them, and then freezing them at (−50 °C) for 24 h. The samples were then filtered through a paper filter using a cooled funnel and the resulting filtrates were placed back in a vacuum oven to evaporate the solvents.

### 3.3. GC-MS Analysis of Cannabinoids in Prepared Extracts

Dry extracts were dissolved in chloroform and analyzed in the presence of an internal standard: diphenylamine dissolved in chloroform at a concentration of 1 mg/mL in a 1:1 ratio. The GC–MS analysis was carried out on Shimadzu QP 2020 Plus apparatus (Shimadzu, Kyoto, Japan) with an installed (Rxi-5ms Restek, Bellefonte, PA, USA) column (30 m × 0.25 mm × 0.25 μm). Then, 1 µL of the mixture was injected at a temperature 250 °C (split ratio 120). The GC oven temperature program started at 180 °C which, after 2 min of hold, was raised to 320 °C at a rate of 35 °C/min. Helium with a linear velocity of 36.3 cm/s was used as the carrier gas.

The results of the analyses were compared to the five-point standard curve prepared according to the above scheme. The NIST 20 database was used in the analyses.

### 3.4. Qualitative Analysis of Terpenoids in Prepared Extracts by HS-SPME-GC-MS

For the qualitative analysis of terpenoid constituents of the obtained extracts, a headspace solid-phase microextraction Arrow (HS-SPME Arrow) method technique with a 1.10 mm DVB/C-WR/ PDMS SPME Arrow fiber (Shimadzu, Kyoto, Japan) was applied. Briefly, 20.0 ± 0.05 g of extracts was placed into 20 mL headspace vials, along with 20 μg of undecane-2-on (Sigma-Aldrich, Steinheim, Germany) as the internal standard. Before the extraction, the samples were incubated for 5 min at 45 °C and, thereafter, the extraction was carried out at the same temperature for 30 min. Then samples were desorbed in a GC–MS apparatus injector for 3 min at 250 °C, with a split ratio of 10. The carrier gas helium 5.0 was used with a linear velocity of 36.3 cm s^−1^. Extractions were performed in three repetitions.

For analyte separation, the Shimadzu GCMS QP 2020 Plus (Shimadzu, Kyoto, Japan) equipped with a Zebron ZB-5 MSi capillary column (30 m × 0.25 mm × 0.25 µm; Phenomenex, Torrance, CA, USA) was used. The following GC program was applied: 50 °C immediately raised to 130 °C with a ratio of 3 °C min^−1^, then to 180 °C with a ratio of 5 °C min^−1^, and then to 280 °C with a ratio of 20 °C min^−1^. The MS operational conditions included an ion source and interface temperature of 250 °C, with a scan mode range of 40–400 *m*/*z*. For qualitative analysis, the experimentally obtained analytes’ mass spectra were compared with the Flavours and Fragrances of Natural and Synthetic Compounds 3.0 (FFNSC 3.0) and National Institute of Standards and Technology (NIST20) libraries. Only scores with a similarity of ≥90 were considered as potential targets. Identification was confirmed by linear retention indices (LRIs), calculated against a C7–C40 mixture of homologous n-alkanes (Sigma-Aldrich, Steinheim, Germany) and restricted to ±15 points. Quantitative analysis was performed by area peak normalization, calculated using the internal standard peak area.

### 3.5. Biological Studies

#### 3.5.1. Cell Lines and Cultured Mediums

Human cell lines: biphenotypic B myelomonocytic leukemia MV4-11, gastric adenocarcinoma AGS, colon adenocarcinoma HT-29, and normal breast MCF-10A cells were obtained from American Type Culture Collection (ATCC) in the United States; lung carcinoma A549 and breast cancer MCF-7 were obtained from European Collection of Authenticated Cell Cultures (ECACC). Human breast cancer MDA-MB-468 cells were obtained from Leibniz Institute DSMZ (German Collection of Microorganisms and Cell Cultures). All the cell lines were maintained at the Hirszfeld Institute of Immunology and Experimental Therapy, PAS, Wroclaw, Poland. MV4-11 and MDA-MB-468 cells were cultured in RPMI 1640 medium (IIET PAS, Wrocław, Poland) with 1.0 mM sodium pyruvate (only MV4-11) and 10% (MV4-11) or 20% (MDA-MB-468) fetal bovine serum (FBS) (all from Merck, Darmstadt, Germany). A549, HT-29, and AGS cells were cultured in RPMI 1640 + Opti-MEM (1:1) medium (IIET PAS, Wrocław, Poland, and Gibco, London, UK) supplemented with 5% FBS (Merck, Darmstadt, Germany) and 1.0 mM sodium pyruvate (only HT-29). The MCF-7 cells were cultured in Eagle medium (IIET PAS, Wrocław, Poland) supplemented with 8 microg/mL of insulin and 1% MEM NON-Essential amino acid (all Merck, Darmstadt, Germany). Normal breast epithelial MCF-10A cells were cultured in the HAM’S F-12 medium, which was supplemented with 10% Horse Serum (Gibco, London, UK), 20 ng/mL EGFh, 10 µg/mL insulin, and 0.5 µg/mL Hydrocortisone and 0.05 mg/mL Cholera Toxin from Vibrio cholerae were from Merck, Darmstadt, Germany.

All culture media were supplemented with 2 mM L-glutamine (Merck, Darmstadt, Germany), 100 units/mL penicillin, (Polfa Tarchomin S.A., Warszawa, Poland), and 100 µg/mL streptomycin (Merck, Germany). All cell lines were grown at 37 °C in a 5% CO_2_ humidified atmosphere.

#### 3.5.2. Determination of Antiproliferative Activity

The antiproliferative activity of cannabinoids (in concentrations of 1 mg/mL in methanol) and the plant extracts named Futu1 and Futu2 (the solutions were prepared by dissolving the substance in methanol to a concentration of 50 mg/mL) were tested against 6 cancer cell lines. Their cytotoxicity was also tested against one normal cell line. The tested mixtures of compounds were diluted in a culture medium to reach the final concentrations. Before adding the tested compounds (24 h prior), the cells were plated in 384-well plates (Greiner Bio-One) at a density of 1 × 10^3^ (A549), 1.5 × 10^3^ (MDA-MB-468, HT-29, MCF-7), or 2 × 10^3^ (AGS, MCF-10A) cells per well. The MV4-11 cells were plated in 96-well plates (Corning) at a density of 5 × 10^3^ cells per well. The assay was performed after 72 h of exposure to 20.0–0.0063 μg/mL concentrations of the cannabinoids and exposure to 200.0–0.063 μg/mL concentrations of Futu1 and Futu2. The in vitro cytotoxic effect of all agents was examined using the MTT (MV4-11) or SRB assay, described previously [42,43]. The results were calculated as the IC_50_ (inhibitory concentration 50%) of the tested agent that was cytotoxic for 50% of the cancer cells. IC_50_ values were calculated for each experiment separately using the Prolab-3 system based on Cheburator 0.4 software [44] and mean values ± SD are presented in Table 1. Each compound in each concentration was tested in triplicate in a single experiment, which was repeated 3–5 times.

## 4. Conclusions

To summarize the results, the impact of extraction methods and parameters on the isolation of cannabinoids and terpenoids from the Futura 75 hemp variety was evaluated. It was demonstrated that the use of binary organic solvent systems enables the efficient isolation of both cannabinoids and terpene fractions from industrial hemp. Additionally, the cannabinoid profile of the extracts was analyzed using gas chromatography coupled with mass spectrometry (GC-MS), while 59 terpenoid compounds were identified via solid-phase microextraction (SPME) combined with GC-MS.

The effects of two selected extracts from the Futura 75 hemp variety—containing cannabinoids and terpene fractions with differing monoterpene-to-sesquiterpene ratios—were investigated for their potential to inhibit cancer cell growth and their safety concerning healthy breast cells. The results indicate that while the extracts are less active than the tested individual cannabinoids, they exhibit significantly higher selectivity toward cancer cells compared to non-tumorigenic cells. Furthermore, the extract with a higher monoterpene content (Futu 2) demonstrated slightly stronger antiproliferative activity.

It was also observed that at lower concentrations, both the individual cannabinoids and the extracts (Futu 1 and Futu 2) stimulated the growth of two cancer cell lines—MV4-11 leukemia cells and the triple-negative breast cancer model MDA-MB-468—while, at higher concentrations, they exhibited an antiproliferative effect. These findings highlight the importance of the extract composition and concentration in correlation with the cancer cell type and line for determining therapeutic efficacy and safety.

## Figures and Tables

**Table 1:** Content of cannabinoid fractions in extracts from the Futura 75 variety obtained by pressure extraction (results given in % by weight).

Solvent	Temp. [°C]	Pressure [bar]	THC % Mean ± SD	CBD % Mean ± SD	CBG % Mean ± SD	CBC % Mean ± SD	CBN % Mean ± SD	% Total Cannabinoids
Ac	25	1	1.10 ± 0.02	42.44 ± 0.51	1.28 ± 0.05	1.96 ± 0.03	1.09 ± 0.02	47.87
		2	1.03 ± 0.02	39.02 ± 0.41	1.16 ± 0.05	1.80 ± 0.02	0.99 ± 0.01	44.00
	−55	1	1.07 ± 0.03	42.65 ± 0.15	1.36 ± 0.08	1.99 ± 0.03	1.11 ± 0.02	48.19
		2	1.01 ± 0.02	39.65 ± 0.39	1.12 ± 0.03	1.85 ± 0.02	1.03 ± 0.01	44.66
MeOH	25	1	0.90 ± 0.05	35.01 ± 0.10	1.35 ± 0.08	1.58 ± 0.12	1.01 ± 0.01	39.85
		2	0.96 ± 0.09	36.84 ± 0.14	1.43 ± 0.12	1.68 ± 0.16	1.11 ± 0.02	42.01
	−55	1	0.79 ± 0.04	34.80 ± 0.03	0.99 ± 0.02	2.02 ± 0.21	1.35 ± 0.03	39.95
		2	0.82 ± 0.04	36.57 ± 0.07	1.03 ± 0.04	2.13 ± 0.28	1.47 ± 0.05	42.02
EtOH	25	1	1.05 ± 0.02	40.03 ± 0.42	1.45 ± 0.03	1.52 ± 0.03	1.11 ± 0.02	45.16
		2	1.24 ± 0.00	42.56 ± 0.56	1.59 ± 0.09	1.63 ± 0.05	1.20 ± 0.00	48.22
	−55	1	0.85 ± 0.03	39.98 ± 0.35	1.05 ± 0.04	1.72 ± 0.06	0.94 ± 0.03	44.54
		2	0.97 ± 0.07	42.98 ± 0.42	1.16 ± 0.05	1.86 ± 0.08	1.03 ± 0.00	48.01
Iso	25	1	0.82 ± 0.02	37.35 ± 0.05	1.35 ± 0.02	1.82 ± 0.10	1.15 ± 0.03	42.49
		2	0.94 ± 0.03	38.25 ± 0.08	1.50 ± 0.05	1.91 ± 0.19	1.33 ± 0.02	43.93
	−55	1	1.02 ± 0.07	36.72 ± 0.38	1.69 ± 0.06	1.82 ± 0.09	1.02 ± 0.01	42.27
		2	1.21 ± 0.20	37.81 ± 0.43	1.91 ± 0.08	1.95 ± 0.15	1.16 ± 0.02	44.03
Hx	25	1	1.35 ± 0.09	43.39 ± 0.09	1.07 ± 0.04	2.10 ± 0.06	1.14 ± 0.03	49.05
		2	1.20 ± 0.07	37.03 ± 0.12	0.95 ± 0.03	1.95 ± 0.02	1.10 ± 0.05	42.23
	−55	1	1.34 ± 0.09	42.92 ± 0.45	0.93 ± 0.05	2.17 ± 0.21	1.25 ± 0.09	48.61
		2	1.15 ± 0.09	36.45 ± 0.28	0.87 ± 0.03	1.89 ± 0.17	1.01 ± 0.03	41.37
Hx:Iso 7:3	25	1	1.21 ± 0.10	43.14 ± 0.75	1.65 ± 0.00	2.08 ± 0.07	1.37 ± 0.02	49.45
		2	1.10 ± 0.05	41.01 ± 0.32	1.45 ± 0.05	1.82 ± 0.10	1.21 ± 0.04	46.59
	−55	1	1.25 ± 0.11	43.87 ± 0.32	1.63 ± 0.05	1.64 ± 0.02	1.10 ± 0.02	49.49
		2	1.05 ± 0.07	40.72 ± 0.21	1.50 ± 0.06	1.52 ± 0.03	1.07 ± 0.03	45.86
Hx:EtOH 7:3	25	1	1.15 ± 0.00	45.96 ± 0.32	1.84 ± 0.09	1.61 ± 0.03	1.02 ± 0.02	51.57
		2	0.95 ± 0.03	42.87 ± 0.39	1.79 ± 0.08	1.56 ± 0.05	0.99 ± 0.01	48.16
	−55	1	1.31 ± 0.32	44.90 ± 0.38	1.69 ± 0.03	1.78 ± 0.03	1.06 ± 0.02	50.74
		2	0.90 ± 0.21	42.01 ± 0.23	1.55 ± 0.02	1.64 ± 0.04	1.01 ± 0.01	47.11

The experiment was conducted in three repetitions (±SD—standard deviation).

**Table 2:** Volatile organic compounds identified in the extract of Futura 75.

Compound	LRI_exp_^1^	LRI_lit_^2^	Content Range Min–Max [%]
α-Pinene	933	933	0.01–1.16
Benzaldehyde	960	960	0.03–0.60
Myrcene	991	991	0.33–11.50
α-Phellandrene	1005	1007	T^3^
Unknown terpene	1010		0.01–0.82
α-Terpinene	1017	1018	0.03–0.87
*o*-Cymene	1024	1025	0.07–10.26
Sylvestrene	1028	1031	T
Eucalyptol	1031	1032	0.01–2.37
γ-Terpinene	1058	1058	0.01–6.59
*cis*-Sabinene hydrate	1067	1069	0.07–0.45
Unknown terpene	1072		0.03–0.43
*p*-Cymenene	1090	1093	0.05–3.36
*trans*-Sabinene hydrate	1099	1099	0.03–1.42
Linalool	1101	1101	0.02–1.76
Fenchol	1114	1119	0.01–3.16
*trans*-Pinene hydrate	1122	1121	0.02–0.88
*trans*-p-Mentha-2,8-diene-1-ol	1136	1140	0.03–0.40
*trans*-Pinocarveol	1139	1141	0.01–0.56
Ipsdienol	1147	1146	0.01–1.82
Unknown terpene	1158		0.03–3.69
Borneol	1166	1173	0.02–2.37
*p*-Cymen-8-ol	1186	1189	2.30–15.23
α-Terpineol	1192	1195	0.12–5.57
Unknown terpene	1198		0.01–0.26
Citronellol	1229	1232	0.01–0.21
α-Ylangene	1374	1371	0.01–2.04
α-Copaene	1378	1375	0.01–0.38
β-Longipinene	1410	1407	T
α-*cis*-Bergamotene	1419	1416	0.01–2.07
E-Caryophyllene	1425	1424	0.02–40.35
β-*cis*-Farnesene	1435	1440	0.01–0.23
α-*trans*-Bergamotene	1440	1432	0.03–13.78
Azulene	1448	1444	0.01–0.24
Alloaromadendrene	1455	1458	T
α-Humulene	1458	1454	4.21–33.50
Sesquisabinene	1461	1455	3.33–17.14
9-*epi*-(*E*)-Caryophyllene	1466	1464	0.01–2.54
γ-Gurjunene	1481	1476	0.01–0.55
α-Amorphene	1484	1482	0.01–0.58
β-Chamigrene	1488	1479	0.01–1.16
β-Selinene	1490	1492	0.01–3.06
Unknown sesquiterpene	1494		0.03–5.24
α-Selinene	1499	1501	0.01–3.36
β-Bisabolene	1512	1508	0.01–5.87
β-Curcumene	1515	1511	0.01–1.06
γ-Cadinene	1518	1512	0.01–0.90
7-*epi*-α-Selinene	1523	1518	T
β-Guaiene	1526	1523	T
Unknown sesquiterpene	1528		0.03–3.32
Selina-4(15),7(11)-diene	1541	1540	0.02–2.98
Unknown sesquiterpene	1543		0.01–1.29
Selina-3,7(11)-diene	1548	1546	0.01–2.58
(*E*)-Nerolidol	1567	1561	0.01–0.78
Caryophyllene oxide	1589	1587	0.02–5.51
Humulene epoxide I	1592	1605	0.01–0.60
Humulene epoxide II	1615	1613	0.01–1.78
Unknown sesquiterpene	1657		0.01–0.49

LRI_exp_^1^—experimentally calculated LRI; LRI_lit_^2^—LRI available in library; T^3^—trace (<0.05%).

**Table 3:** The half maximal inhibitory concentrations (IC_50_) of extracts against selected cancer cell lines and the non-tumorigenic human breast epithelial cell line (MCF-10A) after 72 h of incubation.

Lp	Compound	Cell Lines IC_50_ [μg/mL]				
		A549	HT-29	AGS	MCF-7	MCF-10A
1	THC	3.94 ± 0.44	3.73 ± 0.59	6.35 ± 1.35	8.4 ± 1.26	11.13 ± 0.72
2	CBD	2.87 ± 0.24	3.04 ± 0.52	3.74 ± 0.28	3.67 ± 0.55	10.55 ± 0.63
3	CBG	3.71 ± 0.46	10.04 ± 1.14	6.11 ± 1.31	7.43 ± 1.43	10.74 ± 0.71
4	CBC	3.26 ± 0.20	5.94 ± 1.86	5.53 ± 0.88	7.56 ± 1.52	10.53 ± 0.46
5	CBN	3.30 ± 0.15	6.07 ± 2.11	6.18 ± 0.98	5.79 ± 1.79	10.8 ± 0.68
6	CBDV	6.22 ± 2.26	3.6 ± 0.4	8.4 ± 1.98	8.43 ± 2.05	11.24 ± 0.26
7	Futu1	10.04 ± 1.04	9.92 ± 1.79	15.41 ± 1.55	12.8 ± 2.72	58.0 ± 20.26
8	Futu2	9.34 ± 3.36	7.31 ± 2.24	10.34 ± 2.99	9.01 ± 2.94	31.8 ± 11.25

Data are presented as mean ± standard deviation (SD) calculated using Prolab-3 system based on Cheburator 0.4 software.

**Table 4:** The selectivity index (SI) of tested compounds.

Lp	Compounds	Cell Line/Calculated Selectivity Index SI			
		A549	HT-29	AGS	MCF-7
1	THC	2.82	2.98	1.75	1.33
2	CBD	3.68	3.47	2.82	2.87
3	CBG	2.89	1.07	1.76	1.45
4	CBC	3.23	1.77	1.9	1.39
5	CBN	3.27	1.78	1.75	1.87
6	CBDV	1.81	3.12	1.34	1.33
7	Futu1	5.8	5.85	3.76	4.53
8	Futu2	3.4	4.45	3.08	3.53

The SI index = IC_50_ for normal cell line (MCF-10A)/IC_50_ for respective cancerous cell line. A beneficial SI > 1.0 indicates a drug with efficacy against tumor cells greater than toxicity against normal cells.

## Data Availability

Data are contained within the article and Supplementary Materials.

## Supplementary Materials

The following supporting information can be downloaded at https://www.mdpi.com/article/10.3390/molecules30061325/s1, Table S1. Mass of extract before and after winterization (pressure extraction performed at 1 and 2 bar); Table S2. Mass of primary extracts and after winterization (dynamic extraction, time—10, 20, 40 min.; temp. 25 °C, −55 °C).
