# Supplementary material for: Extraction of Cannabinoids and Terpenes from Hemp Flowers and Leaves (Cannabis sativa L., Futura 75): Chemical Profiling and Evaluation of Anticancer Properties
Source: Molecules. 2025 Mar 15;30(6):1325. doi: 10.3390/molecules30061325 (PMC11946255; doi:10.3390/molecules30061325)
Supplement: Supplementary file 1 [file molecules-30-01325-s001.zip › molecules-3496589-supplementary.pdf]

Supplementary Materials

Extraction of cannabinoids and terpenes from hemp flowers and leaves (*Cannabis sativa* L., Futura 75): Chemical profiling and evaluation of anticancer properties

Monika Haczkiewicz [1], Marta Świtalska [2], Jacek Łyczko [1], Magdalena Pluta [1], Joanna Wietrzyk [2], Anna Gliszczyńska [1,]*[1] Department of Food Chemistry and Biocatalysis, Wrocław University of Environmental and Life Sciences,

Norwida 25, 50-375 Wrocław, Poland; monika.haczkiewicz@upwr.edu.pl (M.H.); magdalena.pluta@upwr.edu.pl (M.P.); jacek.lyczko@upwr.edu.pl (J.Ł.)
[2] Department of Experimental Oncology, Ludwik Hirszfeld Institute of Immunology and Experimental Therapy, Polish Academy of Sciences, Weigla 12, 53-114 Wrocław, Poland; marta.switalska@hirszfeld.pl (M.Ś.); joanna.wietrzyk@hirszfeld.pl (J.W.)
*Correspondence: anna.gliszczynska@upwr.edu.pl (A.G.)

Table S1. Mass of extract before and after winterization (pressure extraction performed at 1 and 2 bar).

Solvent	Temperature [°C]	Pressure [bar]	Mass of extract [g] ± SD	% of mass loss after winterization
Ac	25	1	0.35 ± 0.02	-29%
		2	0.28 ± 0.01	-32%
	-55	1	0.32 ±0.02	-19%
		2	0.27 ± 0.01	-22%
MeOH	25	1	0.41 ± 0.03	-34%
		2	0.46 ± 0.03	-39%
	-55	1	0.26 ± 0.01	-19%
		2	0.29 ± 0.01	-21%
EtOH	25	1	0.33 ± 0.02	-39%
		2	0.37 ± 0.02	-38%
	-55	1	0.29 ± 0.01	-17%
		2	0.32 ± 0.02	-22%
Iso	25	1	0.25 ± 0.00	-36%
		2	0.28 ± 0.01	-36%
	-55	1	0.21 ± 0.00	-19%
		2	0.23 ± 0.00	-22%
Hx	25	1	0.26 ± 0.01	-50%
		2	0.23 ± 0.01	-48%
	-55	1	0.21 ± 0.01	-33%
		2	0.19 ± 0.01	-37%
Hx:Iso (7:3)	25	1	0.32 ± 0.02	-25%
		2	0.27 ± 0.01	-22%
	-55	1	0.28 ± 0.01	-18%
		2	0.26 ± 0.01	-19%

Hx:EtOH (7:3)	25	1	0.29 ± 0.01	-21%
		2	0.26 ± 0.00	-19%
	-55	1	0.23 ± 0.01	-22%
		2	0.21 ± 0.00	-24%

(Ac – acetone, MeOH –methanol, EtOH – ethanol, Iso– isopropanol, Hx – hexane, Hx:Iso (7:3) – hexane:isopropanol (7:3), Hx:EtOH (7:3) – hexane:ethanol (7:3), temperature (25°C and – 55°C)

Table S2. Mass of primary extracts and after winterization (dynamic extraction, time – 10, 20, 40 min.; temp. 25°C, -55°C)

Solvent	Temperature [°C]	Time [min]	Mass [g] ± SD	% mass loss after winterization
Ac	25	10	0.24±0.00	33%
		20	0.30±0.01	37%
		40	0.32±0.01	38%
	-55	10	0.20±0.01	20%
		20	0.22±0.00	23%
		40	0.23±0.00	26%
MeOH	25	10	0.35±0.01	29%
		20	0.41±0.01	29%
		40	0.45±0.01	36%
	-55	10	0.23±0.00	20%
		20	0.25±0.00	20%
		40	0.27±0.00	22%
EtOH	25	10	0.29±0.02	34%
		20	0.34±0.03	44%
		40	0.38±0.01	45%
	-55	10	0.22±0.00	18%
		20	0.23±0.00	22%
		40	0.26±0.00	23%
Iso	25	10	0.25±0.01	32%
		20	0.28±0.02	34%
		40	0.32±0.03	38%
	-55	10	0.21±0.00	19%
		20	0.23±0.00	22%
		40	0.24±0.01	21%
Hx	25	10	0.18±0.00	39%
		20	0.22±0.00	45%
		40	0.25±0.00	48%
	-55	10	0.16±0.00	19%
		20	0.18±0.00	22%
		40	0.20±0.00	20%
Hx:Iso (7:3)	25	10	0.24±0.01	25%

		20	0.26±0.01	31%
		40	0.29±0.01	31%
	-55	10	0.21±0.00	19%
		20	0.23±0.01	22%
		40	0.24±0.00	23%
Hx:EtOH (7:3)	25	10	0.33±0.02	33%
		20	0.34±0.03	32%
		40	0.37±0.03	35%
	-55	10	0.18±0.00	19%
		20	0.19±0.00	21%
		40	0.22±0.00	23%

(Ac – acetone, MeOH –methanol, EtOH – ethanol, Iso – isopropanol, Hx – hexane, Hx:Iso (7:3) – hexane:isopropanol (7:3), Hx:EtOH (7:3) – hexane:ethanol (7:3), temperature (25°C and – 55°C)
